# Optimizing Perioperative Experience in Upper Blepharoplasty: The Impact of Premedication on Anxiety, Pain, and Patient Satisfaction

**DOI:** 10.7759/cureus.53858

**Published:** 2024-02-08

**Authors:** Kristupas A Suslavičius, Laura Liutkauskaitė, Ernest Zacharevskij, Rūta Žuklytė, Medeinė Š Markevičiūtė, Loreta Pilipaitytė

**Affiliations:** 1 Faculty of Medicine, Medical Academy, Lithuanian University of Health Sciences, Kaunas, LTU; 2 Department of Plastic and Reconstructive Surgery, Medical Academy, Lithuanian University of Health Sciences, Kaunas, LTU; 3 Department of Eye Diseases, Republican Vilnius University Hospital, Vilnius, LTU

**Keywords:** anxiety management, local anesthesia, patient satisfaction, pain perception, perioperative experience, ultracod tablets, emla eyelid ointment, benzodiazepines, premedication, upper blepharoplasty

## Abstract

Background

The periorbital area undergoes transformative changes with age, influencing both aesthetic appearance and functional aspects of the eyelids. Age-related alterations involve volume loss, shifts in eyelid crease position, drooping eyebrows, reduced skin elasticity, and the presence of dermatochalasis. Dermatochalasis, characterized by redundant upper eyelid skin folds, poses aesthetic and functional challenges, impacting visual acuity and eyelid elevation efficiency. Upper blepharoplasty addresses these age-related changes. Despite the elective nature of upper blepharoplasty, the procedure can evoke preoperative anxiety and discomfort. Various premedication strategies, including benzodiazepines, aim to alleviate anxiety and enhance the overall patient experience. However, ongoing debates persist regarding the optimal strategy for implementation. The study aims to contribute insights into the effectiveness of different premedication approaches in optimizing patient comfort during and after upper blepharoplasty.

Methods

The research design involves 182 patients divided into three groups: control group (CG) (n = 45) receiving no premedication, Group 1 (n = 98) receiving oral midazolam (a benzodiazepine), and Group 2 (n = 39) receiving a combination of midazolam, eutectic mixture of local anesthetics (EMLA) eyelid ointment, and oral paracetamol with codeine phosphate hemihydrate. The study assesses anxiety levels, pain perception during local anesthetic injection, surgery, and postoperatively, as well as the use of painkillers and adverse effects. Ethical approval was obtained for the study.

Results

Significant differences were noted among the groups during local anesthetic injection (p < 0.0001), surgery (p < 0.0001), and post surgery (p < 0.0197). CG patients experienced higher pain levels during local anesthetic injection and surgery compared to Groups 1 and 2. Group 1 reported more pain during surgery than Group 2. Substantial differences were observed in preoperative (p < 0.0001), during-surgery (p < 0.0001), and after-surgery (p < 0.0001) anxiety levels. The CG exhibited higher preoperative anxiety compared to Group 1, while Group 1 had lower anxiety during surgery compared to the CG. Group 1 also reported lower anxiety after surgery than both the CG and Group 2. A significant difference was found in post-surgery painkiller usage among the groups (p = 0.0003). Group 2 showed significantly lower usage compared to Group 1 (p = 0.0004) and the CG (p = 0.0006). A significant difference was observed in the duration of painkiller use after surgery (p < 0.0014). The CG had a longer duration than Group 1 (p = 0.0049) and Group 2 (p = 0.0495).

Conclusions

Midazolam alone as premedication effectively reduced anxiety before, during, and after surgery. EMLA administration for injection pain did not produce superior results, likely due to its delayed onset. Paracetamol with codeine phosphate hemihydrate effectively reduced surgical pain and postoperative pain duration and decreased the need for painkillers.

## Introduction

The periorbital area undergoes various changes with age, impacting both the appearance and function of the eyelids. These age-related transformations involve the loss of volume in the upper eyelid and periorbital area, a shift in the eyelid crease to a higher position, drooping of the eyebrow (especially its outer part) to a lower position, reduced skin elasticity, and the presence of dermatochalasis [1]. Dermatochalasis, a common condition of the upper eyelid, is characterized by excessive redundant skin folds prolapsing through the weakened orbital septum. This condition can have both aesthetic and functional implications [2]. Dermatochalasis may affect visual acuity, hinder efficient eyelid elevation, and lead to excessive use of the frontalis and orbicular muscles, resulting in discomfort and more prominent forehead wrinkles [3,4]. The primary goal of upper blepharoplasty surgery is to address these age-related changes in the periorbital area.

In 2020, blepharoplasty ranked as the second most popular cosmetic surgery procedure for both women and men, with over 325,000 surgeries performed according to the American Society of Plastic Surgeons. This figure represented an 8% decline from the numbers reported in 2019, attributed to the pandemic-induced reduction in elective surgery rates [5]. Upper blepharoplasty is usually performed under local anesthesia, offering benefits for both the patient and the surgeon [6]. This approach reduces surgical duration and expenses, while also preventing systemic risks associated with general anesthesia and providing faster recovery [6]. Nevertheless, patients may still experience anxiety, discomfort, and psychological distress before and during the procedure.

Anxiety, a natural stress response, manifests as both physical and psychological discomfort [7]. Studies have shown that elevated preoperative anxiety can potentially have adverse effects on both the medical and psychological outcomes of the operation. This includes an increased perception of pain during and after the surgical procedure [7].

Moreover, the injection of local anesthetic often stands out as a painful and distressing event for patients, frequently representing the most unpleasant aspect of the entire blepharoplasty [6,8]. To enhance the patient experience and alleviate the discomfort associated with local anesthetic injection, various combinations of premedication can be employed. Typically, premedication for blepharoplasty involves the administration of medications such as sedatives, anxiolytics, and analgesics before the surgery. The primary goals of these medications are to induce relaxation, reduce anxiety, and manage any potential discomfort during the procedure.

## Materials and methods

Study design

Patients were divided into three groups based on premedication before blepharoplasty. The study excluded participants younger than 18 years old, with a history of smoking and comorbidities such as systemic connective tissue diseases, diabetes mellitus, and ischemic heart disease, and patients with a known allergy to lidocaine or epinephrine. All patients were treated in the plastic and reconstructive surgery department of Kaunas clinics in a day-care setting. Surgeries were performed by two surgeons using the same surgical technique. Patients undergoing blepharoplasty were divided into three groups. The control group (CG) comprised patients who received no premedication. Group 1 comprised patients who got preoperative oral midazolam (a benzodiazepine). Group 2 included patients who received a combination of preoperative oral midazolam, eutectic mixture of local anesthetics (EMLA) eyelid ointment, and oral paracetamol with codeine phosphate hemihydrate. Data for the study were collected through the administration of three English questionnaires. The initial questionnaire was distributed to patients immediately post-blepharoplasty, addressing inquiries regarding anxiety, pain experiences before, during, and immediately after the surgery, as well as the overall surgical experience. The second questionnaire, intended for self-administration at home, prompted patients to report the quantity and timing of painkiller intake, the onset and cessation of postoperative pain, and adverse effects. The third questionnaire was administered seven days post surgery during a follow-up appointment with the operating physician. During this encounter, patients provided feedback on their postoperative experiences after seven days.

Data analysis

The data collected were entered into a Microsoft Excel spreadsheet (Microsoft Corporation, Redmond, WA) and analyzed using GraphPad Prism software version 9.5.1 (GraphPad Software, San Diego, CA). Data normality was assessed using Shapiro-Wilk and Kolmogorov-Smirnov tests. Descriptive statistics were reported using means (±) and median values (min-max) for continuous variables and frequency with percentages for categorical variables. ANOVA, Kruskal-Wallis tests, and post hoc analysis (including Dunnett’s and Dunn’s tests) were selected for the continuous variables. The Pearson chi-squared test or Fisher's exact test was used for categorical variables. All statistical analyses were conducted with two-tailed tests, and a significance level of 0.05 was chosen for hypothesis testing. The study's findings were presented using tables.

Ethical approval

The study was approved by the Bioethics Committee at the Lithuanian University of Health Sciences on December 16, 2022, with approval number BEC-MF-122. All participants were provided with information regarding the study's objectives, and assurances were given regarding the confidentiality of their responses.

## Results

A total of 182 patients (174 females and eight males) met the criteria, with 45 allocated to the CG, 98 to Group 1, and 39 to Group 2. No statistically significant differences were observed in demographic parameters between groups, with a mean age of 56.22 ± 6.476 and a median BMI of 27.61 (19.49-57.14) (Table 1).

**Table 1:** Demographic characteristics

Group	Control	Group 1	Group 2	Total	p-value
Total patients, n	45	98	39	182	
Age, mean (SD)	59.84 (4.587)	56.44 (6.124)	53.97 (8.344)	56.22 (6.476)	<0.0561
Gender, n (%)					0.003
Male	6 (13.33)	1 (1.02)	1 (2.56)	8 (4.40)	
Female	39 (86.67)	97 (98.98)	38 (97.44)	174 (95.60)	
BMI, median (range)	28.57 (20.82-34.29)	27.84 (19.49-40.9)	25.65 (20.34-57.14)	27.61 (19.49-57.14)	0.4644

A significant difference between the three groups was observed in pain sensation during a local anesthetic injection (H(2) = 33.88, p < 0.0001). Post hoc analysis indicated that there was significantly higher pain during local anesthetic injection in the CG than in Group 1 (p < 0.0001) and Group 2 (p < 0.0001). There was no statistically significant difference between Group 1 and Group 2. A significant difference between the three groups was observed in pain sensation during surgery (H(2) = 20.59, p < 0.0001). Dunn’s test revealed that there was higher pain during surgery in the CG than in Group 1 (p = 0.0219) and Group 2 (p < 0.0001). Group 1 felt more pain than those in Group 2 (p = 0.0215). A significant difference between the three groups was observed in pain sensation after surgery. In the CG, pain was higher compared to Group 1 (p = 0.0197), and there was no significant difference between CG and Group 2. Comparisons were conducted among the groups to assess the timing of maximum pain intensity. In both the CG and Group 1, statistically significant increases in pain were observed during local anesthetic injection compared to both during surgery (p < 0.0001) and post surgery (p < 0.0001). In Group 2, a statistically significant reduction in pain was noted during surgery compared to during local anesthetic injection (p < 0.0001) and post surgery (p = 0.0436) (Table 2).

**Table 2:** Pain sensation and feeling of anxiety before, during, and after the operation

Group	Control	Group 1	Group 2	Total	p-value
Pain sensation, median (range)					
During a local anesthetic injection	5 (0-10)	2 (0-7)	2 (0-10)	3 (0-10)	<0.0001
During surgery	2 (0-10)	1 (0-5)	0 (0-5)	1 (0-10)	<0.0001
After surgery	1 (0-10)	1 (0-8)	1 (0-8)	1 (0-10)	0.0223
Feeling of anxiety/stress, median (range)					
In the preoperative period	5 (0-10)	2 (0-10)	4 (0-10)	3 (0-10)	<0.0001
During surgery	3 (0-10)	1 (0-6)	2 (0-8)	2 (0-10)	<0.0001
After surgery	2 (1-10)	0 (0-6)	1 (0-8)	1 (0-10)	<0.0001
Duration of painkillers use in days after surgery, median (range)	2 (1-10)	4 (1-7)	3 (1-4)	3 (1-10)	0.0112
Painkiller consumption in tablets after surgery, median (range)	4 (1-12)	3 (1-28)	3.5 (1-17)	4 (1-28)	0.598

A significant difference between the three groups was observed in feelings of anxiety/stress: in the preoperative period (H(2) = 24.37, p < 0.0001), during (H(2) = 19.13, p < 0.0001), and after the surgery (H(2) = 29.08, p < 0.0001). The post hoc test indicated that anxiety in the preoperative period was significantly higher in the CG compared to Group 1 (p < 0.0001), with no significant difference between the CG and Group 2. Anxiety during surgery was statistically lower in Group 1 than in CG (p < 0.0001), with no significant difference between CG and Group 2. Anxiety after surgery was lower in Group 1 than in CG (p < 0.0001) and Group 2 (p = 0.0058). Comparisons were conducted to examine the peak levels of anxiety within each group. In the CG, statistically significantly elevated anxiety levels were observed during local anesthetic injection compared to both during surgery (p = 0.0108) and after surgery (p < 0.0001). No statistically significant difference was found between anxiety levels during surgery and after surgery. In Group 1, significantly higher anxiety was reported during local anesthetic injection compared to during surgery (p = 0.0075) and after surgery (p < 0.0001). Moreover, statistically significantly higher anxiety was experienced during surgery than after surgery (p < 0.00001). In Group 2, significantly increased anxiety was reported during local anesthetic injection compared to after surgery (p = 0.0004), and no significant difference was observed between local anesthetic injection and during surgery or after surgery (p > 0.05) (Table 2).

A statistically significant difference was observed among the three groups in terms of post-surgery painkiller usage (χ² (2, N = 171) = 16.06, p = 0.0003). Specifically, Group 2 exhibited a significantly lower use of medication compared to Group 1 (p = 0.0004) and CG (p = 0.0006), with no significant difference observed between Group 1 and CG (p > 0.05) (Table 3). A significant difference between the three groups was in the duration of painkillers use in days after surgery (H(2) = 13.17, p < 0.0014). Dunn’s test revealed significantly higher use of painkillers in GC than in Group 1 (p = 0.0049) and Group 2 (p = 0.0495). There was no statistically significant difference in the use of painkiller tablets after surgery between the three groups (p > 0.05) (Table 2).

**Table 3:** Adverse effects, experience after operation, onset, and duration of pain, and postoperative experience after seven days

Group	Control	Group 1	Group 2	Total	p-value
Total patients	45	98	39	182	
Adverse effect(s), n (%)					
Nausea	0 (0.00)	2 (2.15)	2 (5.00)	4 (2.23)	0.2931
Headache	16 (34.78)	9 (9.68)	3 (7.50)	28 (15.64)	0.0002
Tinnitus	0 (0.00)	0 (0.00)	1 (2.50)	1 (0.56)	0.1743
Metallic taste in the mouth	4 (8.70)	1 (1.08)	0 (0.00)	5 (2.79)	0.0178
No adverse effects	26 (56.52)	81 (81.10)	34 (85.00)	141 (78.77)	0.0001
Experience after operation, n (%)					
Went better than I expected	27 (60.00)	63 (64.29)	20 (51.28)	110 (60.44)	0.372
Went as well as I expected	17 (37.78)	35 (35.71)	19 (48.72)	71 (39.01)	0.364
Went worse than I expected	1 (2.22)	0 (0.00)	0 (0.00)	1 (0.55)	0.2164
When started to feel pain after surgery, n (%)					
Never	3 (6.67)	5 (5.38)	14 (42.42)	22 (12.87)	<0.0001
1-2 hours	24 (53.33)	61 (65.59)	9 (27.27)	94 (54.97)	0.0007
3-6 hours	9 (20.00)	20 (21.51)	4 (12.12)	33 (19.30)	0.4974
6-10 hours	6 (13.33)	7 (7.53)	6 (18.18)	19 (11.11)	0.2117
>10 hours	3 (6.67)	0 (0.00)	0 (0.00)	3 (1.75)	0.0139
Duration of pain after surgery, n (%)					
Day of surgery only	1 (2.22)	4 (4.30)	12 (36.36)	17 (9.94)	<0.0001
1 day	21 (46.67)	66 (70.97)	15 (45.45)	102 (59.65)	0.0044
2 days	11 (24.44)	16 (17.20)	3 (9.09)	30 (17.54)	0.2103
3 days	5 (11.11)	6 (6.45)	2 (6.06)	13 (7.60)	0.6667
4 days	2 (4.44)	1 (1.08)	1 (3.03)	4 (2.34)	0.584
5 days	2 (4.44)	0 (0.00)	0 (0.00)	2 (1.17)	0.0588
6 days	3 (6.67)	0 (0.00)	0 (0.00)	3 (1.75)	0.0139
Use of painkillers after surgery, n (%)					0.0003
Yes	36 (80)	71 (76.34)	14 (42.42)	121 (70.76)	
No	9 (20)	22 (23.66)	19 (57.58)	50 (29.24)	
Postoperative experience after 7 days, n (%)					
Went better than I expected	26 (57.78)	63 (67.74)	18 (54.55)	107 (62.57)	0.2996
Went as well as I expected	18 (40.00)	30 (32.26)	15 (45.45)	63 (36.84)	0.3526
Went worse than I expected	1 (2.22)	0 (0.00)	0 (0.00)	1 (0.58)	0.2446

The most common response in each group regarding adverse effects was that participants did not experience side effects. A chi-square and Fisher's exact tests were used to assess homogeneity among the three groups to compare the most common adverse effects on the same day after the operation. Statistically significant differences were observed in headache (χ² (2, N = 179) = 17.29, p = 0.0002), no adverse effects (χ² (2, N = 179) = 18.4, p = 0.0001), and a metallic taste in the mouth (p = 0.0178). When comparing headaches, there was a statistically significant difference, with more instances in the CG than in Group 1 (p = 0.0007) and Group 2 (p = 0.0054), while no significant difference was observed between Group 1 and Group 2 (p > 0.05). Regarding the absence of adverse effects, statistically significantly less were reported in the CG than in Group 1 (p < 0.0001) and Group 2 (p = 0.0041), with no significant difference between Group 1 and Group 2 (p > 0.05). Concerning the metallic taste in the mouth, significantly more patients in the CG reported this sensation after the operation compared to Group 1 (p = 0.0412), while no significant difference was observed between the CG and Group 2, as well as between Group 1 and Group 2 (p > 0.05) (Table 3).

When comparing the groups individually, the most common onset of pain in the CG occurred after one to two hours in Group 1, whereas in Group 2, it was reported as "never." A homogeneity test was conducted to assess the similarity between groups. Statistically significant differences were observed among the three groups regarding the responses "never" and the onset of pain after one to two hours and >10 hours. The occurrence of "never" as a response was significantly higher in Group 2 compared to both CG (p = 0.0002) and Group 1 (p < 0.0001). However, there was no significant difference between the CG and Group 1. Moreover, statistically significantly less pain was reported after one to two hours in Group 2 compared to both CG (p = 0.0214) and Group 1 (p = 0.0001). Regarding pain after >10 hours, statistically significantly more discomfort was reported in the CG compared to Group 1 (p = 0.0331), but no significant difference was observed between the CG and Group 2 (p > 0.05) (Table 3).

Participants in the CG and Group 1 most frequently reported experiencing pain only on the day of surgery, whereas in Group 2, pain was never reported. A homogeneity test was conducted to assess the similarity between groups. There was a statistically significant difference in the duration of pain among individuals who never experienced pain, those who experienced pain solely on the day of surgery, and those who reported pain lasting for five days. When comparing patients who never felt pain, it was significantly more in Group 2 compared to the CG (p < 0.0001) and Group 1 (p < 0.0001). When comparing the pain lasting on the day of surgery, Group 1 exhibited statistically significantly more pain compared to the CG (p = 0.0018) and Group 2 (p = 0.0019), while no significant difference was observed between the CG and Group 2. Comparing the duration of pain among those who experienced it for five days, there was a statistically significant difference, with the CG reporting a longer duration than Group 1 (p = 0.0331), while no significant difference was observed with Group 2 (p > 0.05) (Table 3).

In all three groups, the most common response, both immediately after surgery and after seven days, was that "the operation went better than I expected" (Table 3).

## Discussion

Preoperative anxiety and tension are common concerns for patients undergoing blepharoplasty. Based on the available literature, 38% of patients encounter significant preoperative anxiety before eyelid surgery [7]. To address these challenges, healthcare providers often prescribe oral sedatives, such as benzodiazepines. The use of benzodiazepines as a premedication strategy can notably enhance patient satisfaction with the procedure by reducing their perceived discomfort during needle localization and alleviating anxiety upon entering the operating room. Benzodiazepines target the central nervous system (CNS) to augment the effects of the inhibitory neurotransmitter gamma-aminobutyric acid (GABA), resulting in anxiolysis, sedation, and muscle relaxation [9]. Furthermore, they induce anterograde amnesia, particularly valuable in distressing or painful procedures. With a wide therapeutic index and low toxicity incidence, benzodiazepines further support their use as premedication agents [10].

Midazolam, a fast-acting benzodiazepine with short-term effects, is particularly valued for its ability to swiftly alleviate anxiety and induce drowsiness. Administered buccally, this medication is absorbed through the oral mucosa, offering rapid absorption and a quick onset of action. Due to its unique imidazole ring, midazolam is soluble and stable in water and lipids, allowing for rapid onset and recovery compared to diazepam, attributed to its faster distribution in peripheral tissues and efficient metabolic biotransformation [9,11]. The recommended oral dosage of 0.2-0.5 mg/kg typically results in peak effects within 30-60 minutes of ingestion; hence, it is commonly administered orally one hour before the procedure [9]. The elimination half-life ranges from 1 to 4, with potential prolongation in older individuals; however, cognitive function typically normalizes around four hours post-administration [12]. A randomized, double-blind prospective study involved 150 patients undergoing facial operations, comparing the effects of orally administered midazolam, morphine, and clonidine with a placebo. Although the reduction of anxiety was most prominent in the midazolam (0.15 mg/kg) and clonidine groups, the difference was not statistically significant [13]. Our study, however, revealed a significant reduction in preoperative, intraoperative, and postoperative anxiety among patients who received midazolam. This finding supports the notion that oral benzodiazepines play a crucial role in routine perioperative anxiolysis [10]. According to the literature, the most disadvantageous effect of midazolam as premedication is the lack of pain control. Regarding the severity of pain during the administration of local anesthesia, the midazolam group indicated the highest level of pain (60%), exceeding that of the CG (43%) [13]. However, our study did not confirm such findings.

Pain monitoring in our study was performed using the Visual Analog Scale (VAS), a subjective pain rating system. Participants marked their self-reported pain levels on a 10 cm line ranging from "no pain" at the left end (0 cm) to the "worst pain" at the right end (10 cm). The measurement from the left end to the patient's mark, recorded in centimeters, represented their perceived pain intensity. These values can be utilized to monitor a patient's pain progression or compare pain levels among individuals with similar conditions. Although there are conflicting opinions regarding the superiority of VAS over other pain assessment methods, it continues to be widely used in clinical settings [14].

Decreased sensation from the highly sensitive eyelid skin before injection is one of the few methods to alleviate pain from local anesthetic injection. According to the literature, the application of topical EMLA cream provides a safe and simple way to complement local infiltrative anesthesia in upper blepharoplasty without increasing the risk of complications or adverse effects [15]. EMLA cream consists of 2.5% lidocaine and 2.5% prilocaine in a 1:1 weight ratio. Applied to intact skin, it induces dermal analgesia by accumulating local anesthetics near dermal pain receptors and nerve endings [16]. This preparation significantly reduces skin sensitivity, leading to numbness in the surgical area while retaining the sense of pressure and touch. Consequently, it effectively reduces pain during the administration of anesthetics. Although EMLA cream has demonstrated effectiveness in other body regions, its efficacy for periocular anesthetic injections remains uncertain [6].

For individuals undergoing minor surgery, the administration of local anesthesia is often the most painful aspect of the procedure. The pain results from the skin being pierced, the injected fluid stimulating stretch receptors in the underlying tissues, and the chemical composition of the substance [17]. Our study yielded similar results, with most patients identifying anesthetic administration as the most painful and stressful part of blepharoplasty. However, the intensity of pain was significantly lower in premedicated groups. Additionally, the administration of EMLA and paracetamol with codeine phosphate hemihydrate did not provide superior pain relief. This may be attributed to a significant drawback of topical anesthetics - specifically, the delayed onset of effect. EMLA adequate analgesia is typically achieved one hour after application, reaches its peak at two to three hours, and continues to provide relief for one to two hours after removal [16].

In contrast, skin cooling with ice emerges as a rapid, straightforward, low-risk, and cost-effective method. In a study comparing EMLA and ice, 20 patients received a 0.1 ml injection of lidocaine with adrenaline after the injection site was treated with EMLA cream for 52 minutes or with ice for one to two minutes [18]. Both methods significantly reduced injection pain compared to an untreated area, with EMLA cream only slightly more effective than ice cooling [18]. However, in our study, EMLA and paracetamol with codeine phosphate hemihydrate exhibited the greatest effect during surgery, as patients reported the lowest pain levels compared to those who received benzodiazepine only. It is plausible that the peak effect of premedication occurred at this point.

Paracetamol with codeine phosphate hemihydrate is a compound analgesic containing two active ingredients [19]. Classified within the pharmacotherapeutic group of analgesics, this combination is utilized for the relief of moderate pain [19]. Twelve European Union (EU) member states allow over-the-counter sales of solid dosage forms of codeine, including codeine/paracetamol [20].

Paracetamol, a key component, functions as a pain reliever by likely impeding the generation of impulses at the bradykinin-sensitive chemoreceptors in the periphery that trigger pain [19]. Moreover, paracetamol has been shown to inhibit prostaglandin synthesis more in the CNS than in the periphery, which may account for its limited anti-inflammatory activity [19]. Additionally, it inhibits the effect of endogenous pyrogens on the thermoregulatory center located in the hypothalamus, affecting excitability and causing an antipyretic effect. Codeine, classified as a mild analgesic, acts within the CNS. Its impact is mediated by µ opioid receptors, despite its low affinity for these receptors, and its analgesic properties stem from its transformation into morphine. When combined with other pain relievers like paracetamol, codeine has demonstrated effectiveness in relieving acute nociceptive pain [19].

Postoperative pain, after surgeries under local anesthesia, is a significant concern that has not received enough attention in terms of assessment and treatment. Research indicates that the most severe pain after upper blepharoplasty is experienced in the immediate postoperative period, reaching its peak at 4.4 hours after surgery and lasting up to seven days post surgery [21]. In our study, most of the patients in the CG (53.33%) and midazolam group (65.59%) noted that the sensation of pain appeared one to two hours after the operation. In contrast, among those who received the premedication combination, 42.42% either did not experience pain at all or experienced it later, after six to 10 hours, likely due to exposure to paracetamol with codeine phosphate hemihydrate.

Considering the obtained results, it can be concluded that the post-blepharoplasty pain usually lasts up to two days (94.74%). With combined premedication, the duration of postoperative pain is shorter, as indicated by 81.81% of patients who did not feel pain at all or felt it only on the day of surgery. Also, these patients exhibited a significantly lower use of medication, with 57.58% not requiring painkillers after surgery. In contrast, the majority (80%) of CG patients used pain relievers and continued usage for an extended period, approximately four days. Only in the non-premedicated group did a few patients report pain duration of five or more days.

These results can be explained by the onset times of codeine and paracetamol. Both medications begin to alleviate pain after 30-60 minutes of administration. When administered immediately before surgery, their effects are felt primarily during blepharoplasty. As the analgetic effect lasts for about four to six hours, postoperative pain occurred much later in patients who received this combination, explaining the shortened duration of pain and reduced use of analgesics. This underscores the importance of utilizing paracetamol with codeine phosphate hemihydrate in the preoperative period for effective pain management.

Limitations/weaknesses

There are some limitations to this study. One of the challenges faced was the inability to obtain all study data, as not all patients returned for a follow-up visit. Future investigations should aim to identify specific risk factors associated with increased pain or anxiety, allowing for the prescription of individualized premedication tailored to each patient's unique profile.

## Conclusions

Our study indicates that premedication with only midazolam is an effective tool for reducing anxiety before, during, and after surgery. However, the administration of EMLA to alleviate injection pain did not yield superior results, possibly due to the delayed onset of action. On the other hand, paracetamol with codeine phosphate hemihydrate has emerged as an effective method for significantly reducing pain during surgery, along with decreasing the duration of postoperative pain and the need for analgesics. Recognizing the significance of premedication in upper eyelid blepharoplasty, it becomes evident that tailoring premedication to each patient is crucial for achieving optimal results and enhancing the overall patient experience.
